# On the Deposition of Carbonaceous Matter in the Tissue of the Lungs

**Published:** 1845-11

**Authors:** 


*On the Deposition of Carbonaceous Matter in the Tissue of the Lungs.*—1. There is continually forming and accumulating in the lungs of man, during adult life, and more especially in old age, a certain amount of carbon in a state of the most minute subdivision.

2. This carbon, that exists even in the very substance of the pulmonary tissues, does not come from without.

3. Wherever it exists in sufficient quantity to form deposites of one millimetre in extent, the air-tubes, the blood-vessels, and the pulmonary tissues become transformed into a dark-coloured substance, which may occupy even more than one-half of the entire lungs.

4. The respiration no longer goes on in those parts which serve as a *matrix* to the carbonaceous deposit; there also, the phenomena of the circulation do not take place in the state of disease, and the process of inflammation is consequently never developed.

5. The successive accumulation of this carbon beyond a certain term is apt to cause death in old age, by rendering the pulmonary tissue more or less impermeable to the air.

6. The constant presence of this substance in the lungs of old persons is one cause of the fatality of pneumonia and congestive affections of the respiratory organs in them.

7. These molecules of carbon in the pulmonary parenchyma seem to have a marked influence on the phenomena, which may subsequently occur in and around tuberculous deposits. When tubercles are formed in the lungs, and the carbonaceous matter is deposited in considerable quantity around them, they do not undergo the successive changes proper to phthisis, in the usual course of this disease. The tubercles become calcareous, are deprived of their fatty matter, and do not enlarge. No vessels of new formation are developed around them; or rather, if such vessels have already become enlarged before the deposition of the molecular carbon, they become obliterated in consequence of this deposit, and the progress of the phthisical disease is arrested.

8. The production of carbonaceous matter in the lungs of man,—occurring, as it does, quite independently of any trade or profession, and (most probably) of any particular sort of food—is a fact which should be studied in a pathological as well as a physiological point of view, considering the influence which it may have on the course and issue of the most frequent pulmonic diseases to which old persons are liable. It would seem also that the deposition of this matter in the parenchyma of the lungs has a tendency to arrest the progress of phthisis, by forming a wall around the tubercles, and thus separating them from the intact pulmonary tissue.—*Ibid from Compt. Rend.*

